# A “Notable Address” Indeed

**Published:** 1886-09

**Authors:** 


DANIEL'S

Medical ★ Journal,

PUBLISHED MONTHLY AT

AUSTIN, TEXAS.

F. E. DANIEL, M. D., Editor and Publisher

Subscription Price: Two Dollars per Annum in Advance.

COLLABORATORS.

*E. J. Doering, M. D., Chicago.*
*Geo. Cupples, M. D., San Antonio.*
*Odo Betz, M. D., Germany.*
*E. J. Beall, M. D., Fort Worth, Texas.*
*T. C. Osborn, M. D., Cleburne, "*
*Wm. Penny, M. D., New York.*
*H. O. Marcy, M. D., Boston.*
*C. K. Gregg, M. D., Mexico.*
*R. M. Swearingen, M. D., Austin.*
*C. H. Wilkinson, M. D., Galveston.*
*J. W. McLaughlin, M. D., Austin.*
*R. H. L. Bibb, M. D., Mexico.*

### EDITORIAL.

### A "NOTABLE ADDRESS" INDEED.

"*If thy brother ask thee for a fish, wilt thou give him a stone?*"

In the selection of a successor to Dr. Flint, to deliver the address in Medicine before the British Medical Association, the entire profession of the United States felt that the choice of Dr. John S. Billigs, of the United States Army was a happy one. With a singular unanimity of sentiment the medical press endorsed the selection. The medical profession of the whole country acknowledged Dr. Billings' ability; and knowing and admiring his pleasing address, and versatile talent, felt that the reputation of American medicine would be safe in his hands. The South and West, equally with the North and East, were proud of him. Many thought that he was a great man; at least, that he had the elements of greatness incubating within, and only wanted time and opportunity to develop into a truly great man. Was he not already famous? Was he not already a John Hopkins professor? And many in Europe got an idea that he was the Surgeon General of the United States Army;—just how, we do not know; perhaps his brilliant qualities and distinguished bearing made them think that if he was not, he *ought* to be.

The opportunity came. We looked to see him prove himself worthy of such great anticipations, and the profession was ready to accord to him the first place in American medicine;—Flint's place. Flint's mantle had fallen by common consent upon his shoulders.

This was the opportunity of his life,—the touch-stone of his quality. He had it in his power, had he been truly great, to forever fix the seal of his own fame, and to make secure his title as the greatest of American physicians. Much could have been done to heal the unfortunate breech which he, himself, more than any other, has made in the profession of this country; to re-unite the profession of the sections, which were alienated through unjust discrimination in his appointments. And these things he could have done, not by a sacrifice of veracity,—not by any generosity,—but by *simply being just!*

But it seems the Doctor could not rise above the petty jealousies that characterize the pigmies of medicine. He could not resist the temptation to strike a blow at those who defeated his scheme of organization of the Congress upon a basis which many of the very best men, even of the North, say was not *just*—inasmuch as it was not a national representation. Forgetting every consideration of patriotism and of justice, he stooped to the devices of small souls, and cast a slur upon the profession of the Mississippi Valley.

Dr. Billings has disappointed many. It seems he could not rise to the dignity of a realization of the fact that he was expected to represent AMERICAN MEDICINE, and he stooped to represent a section—a clique! He drew invidious distinctions, and said, in effect, that all the science of the profession resides above, and beyond, the malarial belt! He drew a map, and marked out the sections where malaria prevails,—all throughout the South and West—the great Mississippi Valley. Where malaria is rifest, the map was blackest; and—according to his deductions,—science scarcest.

We will give the doctor credit to suppose that he *meant* the country—the thinly settled region; but he spoke of the Mississippi Valley in its entirety. St. Louis, Memphis, Vicksburg, New Orleans, Chicago—many large cities are in the malarial regions. Did the Doctor really mean to say there are not many physicians in those cities—(not an "exceptional" one)—and even in smaller towns, and even in the country—the peers of most of those north and east? The transactions of the State Medical Association of the "Mississippi Valley States" disprove the assertion!

Dr. Billings has, by this part of his address, brought ridicule, when we expected him to reflect credit. We venture even his British auditors—being familiar with the quarrel over the International Congress, and Dr. Billings' cause for feeling offended—either smiled at what must have seemed a weakness, or *blushed for his effrontery*—blushed, that he should hold up that map and say, "this black spot represents the home of sickness in my country; and it represents, also, and is the limit to the *unenlightened part of the Medical Profession!*"—and this, too, in the presence of the gigantic DAVIS, the Father of Medicine in the Mississippi Valley!

And Flint?—the Great Dead? Ah! he must have groaned aloud in his coffin as HIS SUCCESSOR so slandered twenty thou sand of his constituency!

